# Ventilator settings in ICUs: comparing a Dutch with a global cohort

**DOI:** 10.1186/cc13467

**Published:** 2014-03-17

**Authors:** M Van IJzendoorn, M Koopmans, U Strauch, S Heines, S Den Boer, B Kors, P Van der Voort, P Dennesen, I Van den Hul, E Alberts, P Egbers, A Esteban, F Frutos-Vivar, M Kuiper

**Affiliations:** 1 Medisch Centrum Leeuwarden, the Netherlands; 2 Universitair Medisch Centrum Maastricht, the Netherlands; 3 Spaarneziekenhuis, Hoofdorp, the Netherlands; 4 Kennemer Gasthuis, Haarlem, the Netherlands; 5 Onze Lieve Vrouwe Gasthuis, Amsterdam, the Netherlands; 6 Medisch Centrum Haaglanden, Den Haag, the Netherlands; 7 Vrije Universiteit Medisch Centrum, Amsterdam, the Netherlands; 8 Hospital Universitario de Getafe, Madrid, Spain

## Introduction

In data collected during the Third International Study on Mechanical Ventilation, we compared data from the Netherlands with a global cohort. We hypothesized that tidal volumes (Vt) were smaller and applied positive end-expiratory pressure (PEEP) was higher in the Netherlands, compared with the global cohort. We also compared use of non-invasive ventilation (NIV) and outcomes in both cohorts.

## Methods

A post-hoc analysis of a prospective observational study of patients receiving invasive mechanical ventilation was conducted in 494 ICUs around the world [1]. The Dutch cohort covered 196 patients and the global cohort 7,952.

## Results

Vt was 7.6 ml/kg predicted bodyweight in the Dutch cohort versus 8.0 ml/kg and the median applied PEEP was 8 cmH_2_O versus 5.8 cmH_2_O (both *P *< 0.01). In the subgroup of patients with ARDS, Vt ml/kg was 7.6 and applied PEEP 8.8 cmH_2_O in the Netherlands versus 8.6 ml/kg (*P *= 0.41) and 8.3 cmH_2_O worldwide. In the Netherlands 7.1% of admitted patients received NIV as first mode of mechanical ventilation versus 15% in the global cohort. In both cohorts 18% of patients were hypercapnic at ICU admission. Fewer patients in the Dutch cohort showed an ICU-acquired pneumonia (4.1 vs. 9.4%, *P *= 0. 007) and sepsis (5.1 vs. 9.0%, *P *= 0.042), but more patients evolved delirium (16 vs. 5%, *P *< 0.01).

## Conclusion

According to our hypothesis, Vt was smaller and applied PEEP was higher in the Dutch cohort. Patients in both cohorts received larger Vt than recommended in prevention of ARDS [2]. Hypercapnia is a main criterion for the use of NIV [3], which suggests that NIV could be used more often in the Netherlands. The lower incidence of delirium worldwide could be caused by differences in sedation or may be due to the used methods of screening.
